# Associated Factors for Metabolic Syndrome in the Older Adults with Chronic Virus Hepatitis in the Community

**DOI:** 10.1371/journal.pone.0155544

**Published:** 2016-05-13

**Authors:** Yuan-Hung Kuo, Ming-Chao Tsai, Kwong-Ming Kee, Kuo-Chin Chang, Jing-Houng Wang, Chun-Yin Lin, Sheng-Che Lin, Sheng-Nan Lu

**Affiliations:** 1 Division of Hepatogastroenterology, Department of Internal Medicine, Kaohsiung Chang Gung Memorial Hospital, Kaohsiung, Taiwan; 2 Chang Gung University College Medicine, Taoyuan, Taiwan; 3 Health Center of Yujing district, Tainan, Taiwan; 4 Department of Health, Tainan City Government, Tainan, Taiwan; Chiba University, Graduate School of Medicine, JAPAN

## Abstract

This study was to evaluate the association between metabolic syndrome (MetS) and chronic virus hepatitis elders in the community. Those subjects with positive hepatitis B surface antigen (HBsAg) and/or anti-hepatitis C virus (anti-HCV) screened in the community before were invited to this study and 451 responded. All participants underwent anthropometric measurements, blood tests, ultrasound and fibroscan examinations. The cut-off of liver stiffness measurement-liver cirrhosis (LSM-LC) was 10 kPa for chronic hepatitis B (CHB) patients and 12 kPa for chronic hepatitis C (CHC) patients, respectively. Among 451 responders, 56 were excluded due to negative HBsAg or anti-HCV. Three hundreds and ninety-five subjects included 228 CHB patients, 156 CHC patients and 11 dual hepatitis patients, had a mean age of 62±12.6 years. Fifty-four (23.7%) CHB patients coexisted with MetS whereas 40 (25.6%) CHC patients also had MetS. Those patients with MetS had more LSM-LC cases than those without (20.4% vs 9.8%, p = 0.04 in CHB patients; 28.2% vs 13.5%, p = 0.037 in CHC patients, respectively). In multivariate logistic analysis, detectable viremia was reversely associated with MetS in CHB patients after adjustment for age, gender and body mass index (odds ratio (OR): 0.42; 95% confidence interval (CI): 0.18–0.99; p = 0.047). Regarding CHC patients, higher LSM level was the only factor contributed to MetS (OR: 1.1; 95% CI: 1.02–1.19; p = 0.012). In conclusion, elder CHB patients coexisted with MetS might experience an inactive virus replication but have an advanced liver fibrosis. In elder CHC patients, only higher LSM level was associated with MetS.

## Introduction

Metabolic syndrome (MetS) is a group of metabolic abnormalities including insulin resistance, obesity, hypertension and hyperlipidemia, which is associated with an increased risk of diabetic and cardiovascular mortality[1]. In addition to cardiovascular disease, the presence of MetS is also closely related to other comorbidities such as nonalcoholic fatty liver disease (NAFLD) and its advanced disease, nonalcoholic steatohepatitis (NASH) [2,3]. Indeed, metabolic components of MetS such as obesity and diabetes mellitus (DM) have been proposed as associated risk factors with the development of hepatocellular carcinoma (HCC), especially in those areas where virus hepatitis was not endemic [4]. Since both MetS and chronic virus hepatitis have been reported to be injurious to hepatocytes, the association between MetS and two most virus hepatitis, hepatitis B virus (HBV) and hepatitis C virus (HCV) infection has become the subject of interest in the past decade. Some studies reported MetS coexisted with HCV infection would accelerate insulin resistance, reduce antiviral effect, which leads to a worse outcome [5]. With regard to the interaction of HBV infection and MetS, chronic hepatitis B (CHB) patients were reported to have a lower prevalence of MetS compared with those without [6,7]. The status of HBV replication also seemed to associate with the components of MetS. Moderate-severe hepatic steatosis was reported to contribute to hepatitis B surface antigen (HBsAg) seroclearance in HBsAg carriers [8]. Among hepatitis B e antigen (HBeAg)-positive patients, the REVEAL-HBV study group reported high HBV viral load was inversely associated with obesity [9]. Body mass index (BMI) and HBV viral loads may have synergistic effect on disease progression in HBeAg-negative patients[10].

In Taiwan, the prevalence of MetS is increasing in recent decade [11]. In addition, Taiwan is a HBV-endemic area, and in its southern part, HCV infection is also prevalent [12,13]. Hence, with the increase of MetS population, to realize the relationship between MetS and chronic HBV or HCV infection is necessary in Taiwan. We conducted this community-based study to evaluate the association between MetS and chronic virus hepatitis.

## Methods and Materials

### Patients

In Taiwan, the prevalence of CHB patients and chronic hepatitis C (CHC) patients is 17.3% and 4.4%, respectively [12]. Tainan, located in southern Taiwan, has a high rate of CHC among HCC cases, and the numbers are still increasing [14]. A health examination and cancer screening program was conducted by the Tainan County Health Bureau from April to November 2004. There were 56,702 participants, all of whom were over 40 years of age. Among them, 6133 (10.9%) subjects were positive for HBsAg and 5753 (10.2%) were positive for anti-HCV [15]. A series of HCC screenings were held annually since 2004 to 2010 in the Yujing township and the nearby Nanxi township, located on the mountainside of Tainan had a total of 7643 and 5701 person-times checks, relatively. In Mar 2011, 1027 cases including 671 from Yujing township and 356 from Nanxi township with HBsAg(+) and/or anti-HCV(+) were invited to this study by mail, or telephone. In addition, those volunteers older than 40 years in Yujing township or Nanxi township didn`t be screened before also could participate in this program. All participants underwent anthropometric measurement, blood tests including biochemistry tests and virological tests, ultrasound and fibroscan examinations. Those who with negative value of HBsAg or anti-HCV at the examination were excluded from this study. This study was approved by the Institutional Review Board (IRB) of Kaohsiung Chang Gung Memorial hospital and a written informed consent was obtained from each participant. This study was conducted according to the principles expressed in the Declaration of Helsinki and the consent procedure in the study was also proved by the IRB of Kaohsiung Chang Gung Memorial Hospital.

### Study parameters

We analyzed the participants in terms of demographics (i.e., age and gender), anthropometrics (i.e., body weight, height and waist circumference), hemodynamic measurement (i.e., systolic and diastolic blood pressure), and serum biochemical data (i.e., aspartate aminotransfer (AST), asealanine transaminase (ALT), high-density lipoprotein cholesterol (HDL-C), low-density lipoprotein cholesterol (LDL-C) triglyceride (TG), and fasting glucose levels). This information was obtained from all patients, and they underwent a detailed examination for serum hepatitis markers, including HBsAg, HBeAg, anti-HCV, HBV DNA, HCV RNA and alpha-fetoprotein (AFP). HBsAg, HBeAg and anti-HCV were detected using commercial assay kits (HBsAg EIA, Abbott, North Chicago, IL; HBeAg EIA, Abbott; anti-HCV, EIA 3.0, Abbott). Levels of HBV DNA were measured by the Cobas Taqman assay (Roche Diagnostics, Branchburg, NJ, USA), with a lower limit of detection of 12 IU/mL. Levels of HCV RNA were detected using a Cobas AmpliPrep/Cobas TaqMan HCV kit (Amplicor, Roche Diagnostics, Branchburg, NJ), with a lower limit of detection of 15 IU/ml.

### Liver stiffness measurement

Liver stiffness measurement (LSM) was performed with a fibroScan_ (Echosens, Paris, France) system, a device based on one-dimensional transient elastography technique. The details of the technical background and examination procedure have been previously defined [16]. The result was considered reliable only when 10 validated measurements had been obtained with a success rate greater than 60%.The cut-off of liver stiffness measurement-liver cirrhosis (LSM-LC) was 10 kPa for chronic hepatitis B (CHB) patients and 12 kPa for chronic hepatitis C (CHC) patients, respectively.

### Definition of Metabolic syndrome

MetS was defined as the fulfillment of at least three of the following criteria: (1) abdominal obesity (waist: males ≧90 cm, females ≧ 80 cm); (2) blood pressure [systolic pressure (SBP) ≧ 130 mmHg or diastolic pressure (DBP) ≧85 mmHg]; (3) hyperglycemia [fasting glucose (FG) ≧ 100 mg/dL]; (4) HDL-C (males < 40 mg/dL, females < 50 mg/dL); and (5)TG ≧ 150 mg/dL [17].

### Statistical analysis

Average values are expressed as mean±SD. The logistic regression analysis model was used to estimate the univariate and multivariate effects of the different risks regard to the incidence of MetS. Odds ratios (OR) and 95% confidence intervals (CI) were used to describe the strength of the association between risk factors and MetS. A p value <0.05 was considered statistically significant.

## Results

**Fig 1:**
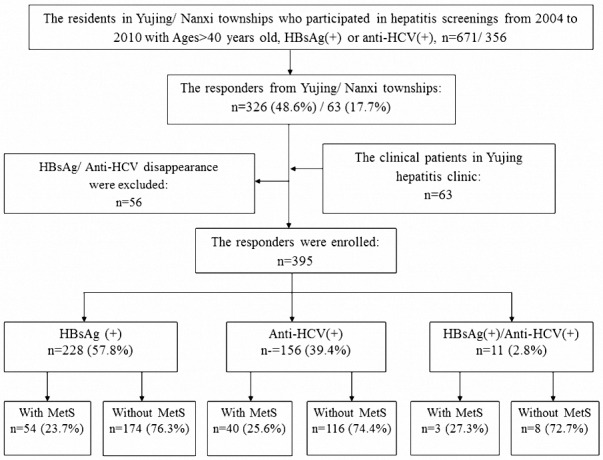
The flow chart of the chronic hepatitis patients enrolled into this study. MetS represents metabolic syndrome.

**Fig 2:**
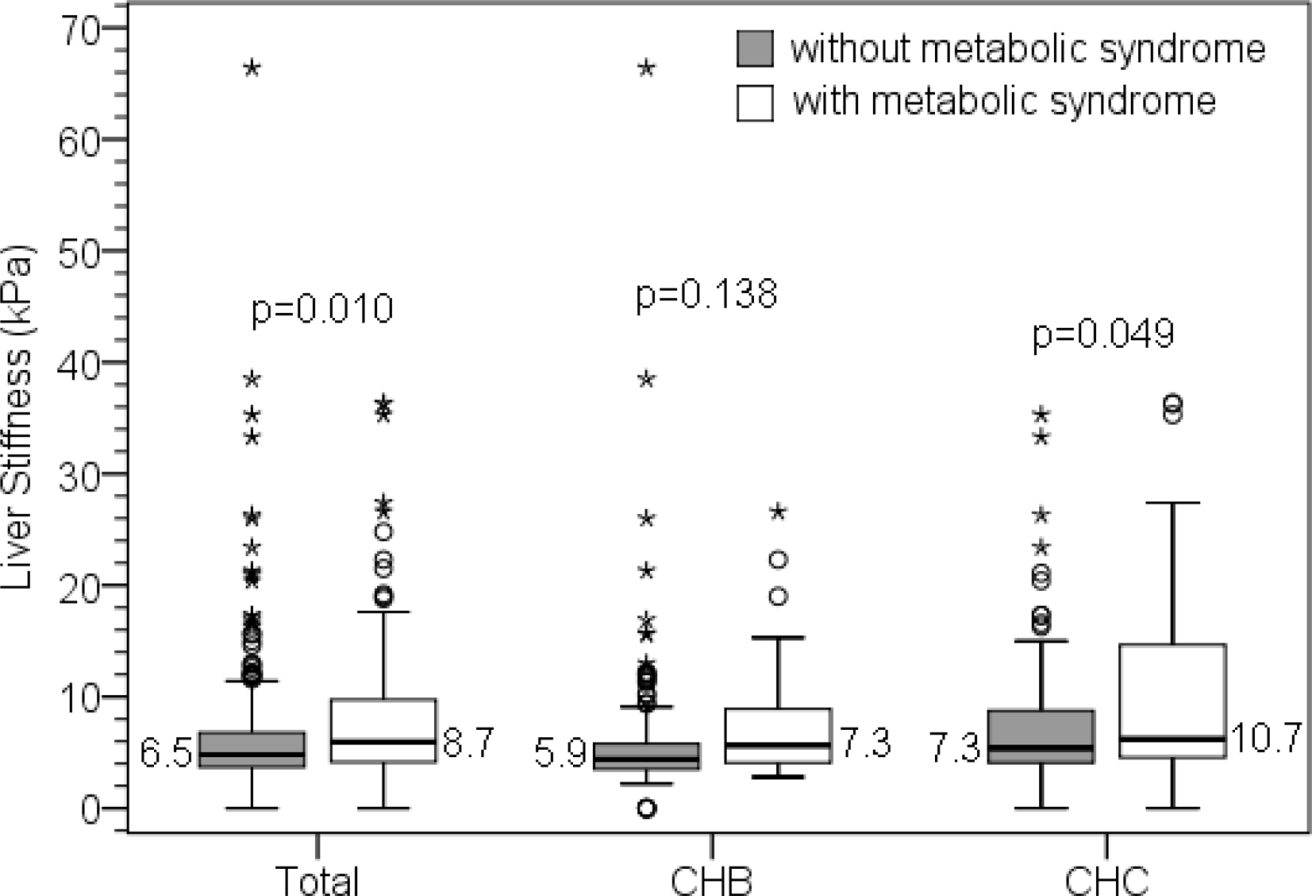
Total patients and chronic hepatitis C (CHC) patients coexisted with MetS had significant higher liver stiffness level than those without (8.7kPa vs 6.5 kPa, p = 0.01; 10.7 kPa vs 7.3 kPa, p = 0.049, respectively). Although chronic hepatitis B (CHB) patients coexisted with Mets also had higher liver stiffness level than those without, the difference was not significant (7.3 kPa vs 5.9 kPs, p = 0.138).

Among 451 responders, 326 subjects and 63 subjects were from previous Yujing and Nanxi community hepatitis screenings, respectively; the other 62 subjects were the volunteers in Yuing township. After excluding 56 subjects with the negative finding of HBsAg or anti-HCV tests at this baseline examination, a total of 395 subjects were enrolled. (Fig 1) They included 179 men and 216 women with a mean age of 62±12.6 years. Among them, 228 (57.7%) subjects were positive of HBsAg, 156 (39.5%) were positive of anti-HCV, and another11 (2.8%) were both positive, respectively. Ninety-seven subjects (24.6%) fitted the criteria of MetS. CHC patients were older, had higher ALT, AST, and LSM levels as well as had more DM cases than CHB patients. But in the ratio of MetS cases and the value of BMI, there was no difference between the two groups. (Table 1) Those chronic hepatitis patients with MetS had a significant higher LSM level than those without (8.7±7.45 vs 6.5±6.14 kPa, p = 0.01). (Fig 2) Among our CHB or CHC patients, those who with MetS had higher LSM level than those without, but the difference in CHB patients was not significant. However, regarding the LSM-LC cases, CHB and CHC patients coexisted with MetS had more cases than those without (20.4% vs 9.8%, p = 0.04 in CHB patients; 30% vs 16.4%, p = 0.037 in CHC patients, respectively). (Tables 2 and 3)

**Table 1:** Baseline characteristics of enrolled 395 patients.

	CHB	CHC	B+C	P-value*
	n = 228	n = 156	n = 11	
Age, mean±SD (y/o)	57.8± 11.4	67.5±12.7	57.5±12.3	0.166
Male sex, n(%)	108(47.4)	68(43.6)	3(27.3)	0.465
AST(IU/L), median (IQR)	26(22–33)	34.5(25–55)	32(26–103)	<0.001
ALT(IU/L), median (IQR)	24(18–35.3)	31(22–60.5)	38(17–99)	0.001
AFP(ng/mL), median (IQR)	2.5(1.89–3.8)	3.2(2.2–4.9)	2.2(1.74–2.8)	<0.001
LSM (kPa), mean±S.D	6.2± 6.0	8.2± 7.2	7.4 ± 4.8	0.001
BMI (kg/m2)	24.7 ± 3.8	24 ± 3.3	23.8 ± 4.7	0.164
MetS, n(%)	54(23.7)	40(25.6)	3(27.3)	0.661
DM, n(%)	16(7.3)	26(17.3)	0	0.003
US_LC, n(%)	22(9.9)	23(15.2)	0	0.121
LSM_LC, n (%)	28(12.3)	31(20.7)	1(9.1)	0.029

Abbreviations: n:numbers; CHB: chronic hepatitis B; CHC: chronic hepatitis C: B+C: chronic hepatitis B plus chronic hepatitis C; AST: aspartate aminotransferase

ALT: Alanine Aminotransferase; LSM: liver stiffness measurement; HCC: hepatocellular carcinoma; DM: diabetes mellitus; MetS: metabolic syndrome; US_LC: untrasound diagnosed liver cirrhosis; LSM_LC: liver stiffness measurement diagnosed liver cirrhosis; BMI: body mass index

*Comparison between CHB and CHC patients.

**Table 2:** Baseline characteristics of enrolled 228 CHB patients.

	With MetS,	Without MetS,	P-value
	n = 54	n = 174	
Age (year), mean±S.D	59.8± 10.3	57.2± 11.8	0.143
Male sex, n(%)	23(42.6)	85(48.9)	0.421
AST(IU/L), median (IQR)	27 (22–33)	26 (22–33	0.192
ALT(IU/L), median (IQR	27 (18.8–43.5)	24 (17–34)	0.302
AFP(ng/mL), median (IQR)	2.77 (2.03–3.9)	2.49 (1.9–3.73)	0.847
LSM (kPa), mean±S.D	7.3± 5	5.9± 6.3	0.138
Log (HBV DNA)	2.8± 1.7	3.3± 1.6	0.055
BMI (kg/m^2^) mean±S.D	26.9 ± 3.6	23.9 ± 3.6	<0.001
DM, n(%)	10(18.5)	6(3.7)	<0.001
DNA<12 IU/mL*,n(%)	13(24.1)	21(12.1)	0.033
US_LC, n(%)	5(9.4)	17(10.1)	0.894
LSM_LC, n(%)	11(20.4)	17(9.8)	0.04

*The limit of detectable HBV DNA level is 12 IU/mL.

Abbreviations: CHB: chronic hepatitis B; AST: aspartate aminotransferase

ALT: Alanine Aminotransferase; AFP: alpha-fetoprotein; LSM: liver stiffness measurement; BMI: body mass index; DM: diabetes mellitus; MetS: metabolic syndrome; US-LC: untrasound diagnosed liver cirrhosis; LSM_LC: liver stiffness measurement diagnosed liver cirrhosis.

**Table 3:** Baseline characteristics of enrolled 150 CHC patients.

	With MetS,	Without MetS,	P-value
	n = 40	n = 116	
Age (year), mean±S.D	67± 17.9	67.6± 10.3	0.795
Male sex, n(%)	14(35)	54(46.6)	0.204
AST(IU/L), median (IQR)	35 (23.8–74.8)	34.5 (25–53)	0.638
ALT(IU/L), median (IQR)	35.5 (24.3–63)	31 (21–60.5)	0.743
AFP(ng/mL), median (IQR)	4.16 (2.7–7.3)	3 (2.1–4.7)	0.027
LSM (kPa), mean±S.D	10.7± 9.8	7.3± 5.9	0.049
Log (HCV RNA)	4.4± 2.2	4.2± 2.2	0.548
BMI (kg/m^2^) mean±S.D	25 ± 3.4	23.7 ± 3.2	0.024
DM, n(%)	15(38.5)	11(9.9)	<0.001
RNA<15 IU/mL*, n(%)	10(25)	30(25.9)	0.248
US_LC, n(%)	8(21.1)	15(13.3)	0.089
LSM_LC, n(%)	12(30)	19(16.4)	0.037

*The limit of detectable HCV RNA level is 15 IU/mL.

Abbreviations: CHC: chronic hepatitis C; ALT:Alanine Aminotransferase;AST: aspartate aminotransferase; AFP: alpha-fetoprotein; LSM: liver stiffness measurement; BMI: body mass index; DM: diabetes mellitus; MetS: metabolic syndrome; US-LC: untrasound diagnosed liver cirrhosis; LSM_LC: liver stiffness measurement diagnosed liver cirrhosis.

Regarding the viremia, these elder CHB patients coexisted with MetS had more undetectable viremia cases than those without MetS (24.1% vs 12.1%, p = 0.033). (Table 2) However, the comparison of RNA level between our CHC patients with or without MetS was insignificant. (Table 3)

In the multiple logistic regression analysis, higher liver stiffness level was still associated with the presence of MetS in our chronic hepatitis patients after adjustment for age, gender and body mass index (odds ratio (OR): 1.08; 95% confidence interval (CI): 1.01–1.14; p = 0.016) (Table 4). We further focused on the CHB patients, the associated factors with MetS were older age, female sex, undetectable viremia, and higher BMI value in the multiple logistic regression analysis. Whereas in the CHC patients, only higher LSM level contributed to the presence of MetS in both univariate and multivariate logistic regression analysis. Although higher BMI value was also a significant variable related to MetS in the univariate analysis, the impact was diluted after adjustment for the other variables.

**Table 4:** Logistic regression analysis of factors associated with metabolic syndrome for enrolled total patients, chronic hepatitis B patients and chronic hepatitis C patients.

	Univariate		Multivariate	
Total patients, n = 395				
	OR (95% CI)	P-value	OR (95% CI)	P-value
Age	1.01(0.99–1.03)	0.232	1.06(1.03–1.09)	<0.001
(per one year increase)				
Male vs female	0.68(0.42–1.08)	0.103	0.45(0.26–0.78)	0.005
Hepatitis				
HBV	1			
HCV	1.21(0.31–4.72)	0.785		
HBV+HCV	1.11(0.69–1.78)	0.661		
US-LC				
Positive vs Negative	0.77(0.39–1.54)	0.465		
Serum AST (IU/ml)				
40 vs <40	1.32(0.8–2.18)	0.271		
Serum ALT (IU/ml)				
40 vs <40	1.47(0.9–2.4)	0.124		
(DNA-RNA), n(%)				
(-, -), 140 (31)	1			
(-,+), 107 (23.7)	0.77(0.41–1.47)	0.426		
(+,-), 199 (44.1)	0.67(0.37–1.18)	0.166		
(+,+), 5 (1.1)	3.6(0.57–22.9)	0.175		
Liver stiffness	1.05(1.01–1.08)	0.008	1.08(1.01–1.14)	0.016
(per 1 kPa increase)				
Body mass index	1.2(1.12–1.29)	<0.001	1.24(1.14–1.34)	<0.001
(per 1 kg/m^2^ increase)				
CHB patients, n = 228				
	OR (95% CI)	P-value	OR (95% CI)	P-value
Age	1.02(0.99–1.05)	0.144	1.06(1.02–1.09)	0.002
(per one year increase)				
Male vs female	0.78(0.42–1.44)	0.422	0.44(0.21–0.91)	0.026
US-LC				
Positive vs Negative	0.93(0.33–2.66)	0.894		
Serum AST (IU/ml)				
40 vs <40	1.37(0.63–2.99)	0.424		
Serum ALT (IU/ml)				
40 vs <40	1.62(0.8–3.28)	0.181		
HBV DNA				
detectable vs undetectable	0.44(0.20–0.95)	0.037	0.42(0.18–0.99)	0.047
Liver stiffness	1.03(0.99–1.08)	0.17		
(per 1 kPa increase)				
Body mass index	1.25(1.14–1.36)	<0.001	1.28(1.15–1.42)	<0.001
(per 1 kg/m^2^ increase)				
CHC patients, n = 156				
	OR (95% CI)	P-value	OR (95% CI)	P-value
Age	0.99(0.97–1.02)	0.794		
(per one year increase)				
Male vs female	0.62(0.29–1.30)	0.206		
US-LC				
Positive vs Negative	1.74(0.67–4.51)	0.252		
Serum AST (IU/ml)				
40 vs <40	1.13(0.55–2.32)	0.75		
Serum ALT (IU/ml)				
40 vs <40	1.4(0.68–2.91)	0.364		
HCV RNA				
detectable vs undetectable	1.1(0.48–2.53)	0.825		
Liver stiffness	1.06(1.01–1.11)	0.018	1.1(1.02–1.19)	0.012
(per 1 kPa increase)				
Body mass index	1.14(1.02–1.28)	0.027		
(per 1 kg/m^2^ increase)				

Abbreviations: MetS: metabolic syndrome; CHB: chronic hepatitis B; CHC: chronic hepatitis C; OR: odds ratio; CI: confidence interval; US-LC: untrasound diagnosed liver cirrhosis; AST: aspartate aminotransferase; ALT:Alanine Aminotransferase;HBV: hepatitis B virus; HCV: hepatitis C virus; vs: versus.

## Discussion

The current study showed that among the elders in the community, there were 23.7% MetS subjects in CHB patients and 25.6% in CHC patients, respectively. The ratio of MetS subjects was significant higher than another large population-based study reported by Jan et al in northern Taiwan [6]. which showed 8% in CHB patients and 13.2% in CHC patients, respectively. This difference was resulted from the age variations of the selected study cohorts. The current study had 82.3% subjects older than 50 years whereas only 51.1% in the Jan et al`s study. Indeed, older age was an independent factor associated with the presence of MetS after adjustment for gender, LSM and BMI level in this study.

Liver stiffness level has been identified to represent the severity of liver fibrosis. The current study found that higher liver stiffness level was associated with the presence of MetS. Our subjects with MetS had a significant higher LSM level than those without (8.69±7.45 vs 6.49±6.14 kPa, p = 0.002), which was comparable with the previous studies (18,19). Roulot D et al reported that in 429 healthy subjects, after adjustment for gender and BMI, LSM values were higher in 59 (13.7%) subjects with MetS than in those without. (6.51 ± 1.64 vs 5.33 ± 1.51 kPa, p < 0.0001) [18]. Mena A et al also reported that in 96 CHB inactive carriers who with MetS had a higher LSM value than those without. (8.4kPa vs 5.5 kPa, p<0.001) [19]. The presence of MetS was associated with higher LSM level not only in chronic hepatitis patients but in health subjects and inactive CHB carriers.

The interaction of different components of MetS and CHB infection has been studied in recent decade. A Hong Kong study showed that HBV infection was associated with a lower prevalence of MetS [7], which was comparable with the observation in Taiwan [6]. Our previous study found that HBsAg carriers had lower prevalence of hypercholesterolemia and hypertriglyceridemia [20]. The current study also indicated that the inverse association between HBV viral load and MetS. In our CHB patients, with the increase of viral load, the ratio of MetS subjects was decreased with a significant lineal trend (p = 0.024). Undetectable viremia was associated with the presence of MetS after adjustment for age, gender and BMI.

Although the presence of MetS seems to be beneficial to the decrease of HBV activity, the causal relationship is undetermined. In fact, we found that MetS was also related to the poor prognosis of HBV infection such as advanced fibrosis. In our previous study, LSM level more than 10 kPa was used to predict cirrhosis for HBsAg carrier with a negative predictive value of 95% [16]. The current study showed that HBsAg carriers with MetS had more LSM-LC cases than those without. Regarding HBV related HCC, the association with MetS was undetermined. Our previous longitudinal community study reported that MetS, and obesity were not associated with HCC development in HBV patients after a 4 year observation [21]. However, another longitudinal cohort study in Taiwan indicated that, during mean follow-up of 14.7 years, excess body weight was involved in the transition from health HBV carrier state to HCC or liver-related death among men [22]. The variation associated the impact of MetS on HBV related HCC might be resulted from the different follow-up years.

Our CHB subjects were older with a mean age of 57.8± 11.4 years. Among 228 CHB subjects, even 75% were older than 50 years and located in the inactive residual phase of HBV nature history [23]. In elder CHB subjects, we found the presence of MetS was associated with undetectable HBV viremia. However, CHB subjects coincided with MetS had severer liver fibrosis than those without. The current study indicated that the elders in the later phase of HBV infection coincided with MetS might experience an inactive virus replication but have an advanced liver fibrosis than those without. The effect of MetS between reducing HBV activity and inducing hepatocyte inflammation should be balanced.

The review article indicated that HCV infection does not carry an increased risk of MetS, but it could disturb glucose homeostasis, leading to both intrahepatic and extrahepatic insulin resistance [24]. This reduced the response to antiviral treatment and accelerated liver disease progressions such as the advance of cirrhosis or the development of HCC. The current study showed that HCV viremia was not related to the presence of MetS. Only higher LSM level contributed to MetS in CHC patients in multivariate logistic analysis. Although higher BMI value was also associated with MetS in univariate logistic analysis, the impact was diluted after adjustment for the other variables. Based on our previous study, 12 kPa was the cut-off for the prediction of possible cirrhosis in CHC patients [16]. The current study showed that CHC patients with MetS also had more LSM-LC cases than those without. This result suggested that no matter in CHB patients or CHC patients, MetS showed a significant association with an advanced liver fibrosis and might lead to an unfavorable outcome.

We also found that in our CHB or CHC patients, the difference between those who with or without MetS was not significant when evaluated by US but it was significant when evaluated by LSM. It seems to be related to the increase of patients diagnosed as LC by LSM in MetS patients. Recent studies showed that the severity of liver fibrosis might be overestimated by LSM in the presence of severe steatosis in patients with CHC or with nonalcoholic fatty liver disease, which might explained the difference of LC evaluated by US and by LSM in our study [25,26].

The study had certain limitations. First, although we found MetS had the inverse association with HBV viremia, the causal relationship was undetermined by a cross-sectional analysis. A longitudinal observation should be required to clarify this issue. Furthermore, central obesity, the main component of MetS is the usual determinant of LSM failure or unreliable results [27]. For those extreme obesity patients with particularly increased waist circumference or narrowing intercostal space, the result of LSM might be slightly doubted.

## Conclusion

These results suggest that the elders in the later phase of HBV infection coincided with MetS might experience an inactive virus replication but have an advanced liver fibrosis than those without. Among our CHC subjects, MetS was associated with higher LSM level, which means the presence of advanced liver fibrosis. Hence, to the elders with CHB or CHC infection, the clinicians should endeavor to eliminate the components of MetS to diminish the harmful effect of liver steatosis.

## References

[1] Eckel RH, Alberti KG, Grundy SM, Zimmet PZ. The metabolic syndrome. Lancet 2010;375:181–3. 10.1016/S0140-6736(09)61794-3 20109902

[2] Nugent C, Younossi ZM. Evaluation and management of obesity-related nonalcoholic fatty liver disease. Nat Clin Pract Gastroenterol Hepatol 2007;4:432–41. 17667992 10.1038/ncpgasthep0879

[3] Vuppalanchi R, Chalasani N. Nonalcoholic fatty liver disease and nonalcoholic steatohepatitis: selected practical issues in their evaluation and management Hepatology 2009;49:306–17. 10.1002/hep.22603 19065650 PMC2766096

[4] Jinjuvadia R, Patel S, Liangpunsakul S. The association between metabolic syndrome and hepatocellular carcinoma: systemic review and meta-analysis. J Clin Gastroenterol. 2014;48(2):172–7. 10.1097/MCG.0b013e3182a030c4 24402120 PMC3887366

[5] Negro F. Steatosis and insulin resistance in response to treatment of chronic hepatitis C. J Viral Hepat. 2012;19:43–7. 10.1111/j.1365-2893.2011.01523.x 22233413

[6] Jan CF, Chen CJ, Chiu YH, Chen LS, Wu HM, Huang CC, et al A population-based study investigating the association between metabolic syndrome and hepatitis B/C infection (Keelung Community-based Integrated Screening study No. 10). Int J Obes (Lond). 2006;30(5):794–9. 16404404 10.1038/sj.ijo.0803204

[7] Wong VW, Wong GL, Chu WC, Chim AM, Ong A, Yeung DK, et al Hepatitis B virus infection and fatty liver in the general population. J Hepatol. 2012;56(3):533–40. 10.1016/j.jhep.2011.09.013 22027575

[8] Chu CM, Lin DY, Liaw YF. Does increased body mass index with hepatic steatosis contribute to seroclearance of hepatitis B virus (HBV) surface antigen in chronic HBV infection? Int J Obes (Lond). 2007;31(5):871–5. 17047638 10.1038/sj.ijo.0803479

[9] Chiang CH, Yang HI, Jen CL, Lu SN, Wang LY, You SL, et al REVEAL-HBV Study Group. Association between obesity, hypertriglyceridemia and low hepatitis B viral load. Int J Obes (Lond). 2013;37(3):410–5. 22531094 10.1038/ijo.2012.63

[10] Lee IC, Huang YH, Chan CC, Huo TI, Chu CJ, Lai CR, et al Impact of body mass index and viral load on liver histology in hepatitis B e antigen-negative chronic hepatitis B. Clin Nutr. 2011;30(5):647–52. 10.1016/j.clnu.2011.05.001 21612848

[11] Huang KC. Obesity and its related diseases in Taiwan. Obes Rev 2008; 9 (Suppl 1):32–4. 10.1111/j.1467-789X.2007.00435.x 18307696

[12] Chen CH, Yang PM, Huang GT, Lee HS, Sung JL, Sheu JC. Estimation of seroprevalence of hepatitis B virus and hepatitis C virus in Taiwan from a large-scale survey of free hepatitis screening participants. J Formos Med Assoc 2007;106:148–55. 17339159 10.1016/S0929-6646(09)60231-X

[13] Tung HD, Wang JH, Tseng PL, Hung CH, Kee KM, Chen CH, et al Neither Diabetes Mellitus nor Overweight Is a Risk Factor for Hepatocellular Carcinoma in a Dual HBV and HCV Endemic Area: Community Cross-Sectional and Case–Control Studies. Am J Gastroenterol 2010;105;624–31. 10.1038/ajg.2009.711 20051944

[14] Chang KC, Lu SN, Chen PF, Hung CH, Kee KM, Yen YH, et al Incidence and associated risk factors of hepatocellular carcinoma in a dural hepatitis B and C virus endemic area: A surveillance study. Kaohsiung J Med Sci. 2011:27:85–90. 10.1016/j.kjms.2010.11.001 21421195 PMC11916633

[15] Tsai MC, Kee KM, Chen YD, Lin LC, Tsai LC, Lu SN. Excess mortality of hepatocellular carcinoma and morbidity of liver cirrhosis and hepatitis in HCV-endemic areas in an HBV-endemic country: Geographic variations among 502 villages in southern Taiwan. J Gastroenterol Hepatol 2007;22:92–8. 17201888 10.1111/j.1440-1746.2006.04489.x

[16] Wang JH, Changchien CS, Hung CH, Eng HL, Tung WC, Kee KM, et al FibroScan and ultrasonography in the prediction of hepatic fibrosis in patients with chronic viral hepatitis. J Gastroenterol 2009;44:439–46. 10.1007/s00535-009-0017-y 19308312

[17] Alberti KG, Zimmet P, Shaw JIDF Epidemiology Task Force Consensus Group. The metabolic syndrome—a new worldwide definition. Lancet 2005;365:1059–62. 10.1016/S0140-6736(05)67402-8 16182882

[18] Roulot D, Czernichow S, Le Clésiau H, Costes JL, Vergnaud AC, Beaugrand M. Liver stiffness values in apparently healthy subjects: influence of gender and metabolic syndrome. J Hepatol. 2008;48(4):606–13. 10.1016/j.jhep.2007.11.020 18222014

[19] Mena A, Pedreira JD, Castro A, López S, Vázquez P, Poveda E. Metabolic syndrome association with fibrosis development in chronic hepatitis B virus inactive carriers. J Gastroenterol Hepatol. 2014;29(1):173–8. 10.1111/jgh.12432 24219115

[20] Chen CY, Wang JH, Lin CY, Chen PF, Tseng PL, Chen CH, el al Lower prevalence of hypercholesterolemia and hyperglyceridemia found in subjects with seropositivity for both Hepatitis B and C strains independently. J Gastroenterol Hepatol 2010;25:1763–8. 10.1111/j.1440-1746.2010.06300.x 21039839

[21] Chen CT, Chen JY, Wang JH, Chang KC, Tseng PL, Kee KM, et al Diabetes mellitus, metabolic syndrome and obesity are not significant risk factors for hepatocellular carcinoma in an HBV- and HCV-endemic area of Southern Taiwan. Kaohsiung J Med Sci. 2013:29:451–9. 10.1016/j.kjms.2012.12.006 23906236 PMC11916133

[22] Yu MW, Shih WL, Lin CL, Liu CJ, Jian JW, Tsai KS, et al Body-mass index and progression of hepatitis B: a population-based cohort study in men. J Clin Oncol. 2008;26(34):5576–82. 10.1200/JCO.2008.16.1075 18955457

[23] Chu CM, Liaw YF. Hepatitis B virus infection. Lancet 2009:373:582–92. 10.1016/S0140-6736(09)60207-5 19217993

[24] Bugianesi E, Salamone F, Negro F. The interaction of metabolic factors with HCV infection: does it matter? J Hepatol. 2012;56 Suppl 1:S56–65. 10.1016/S0168-8278(12)60007-5 22300466

[25] Macaluso FS, Maida M, Cammà C, Cabibbo G, Cabibi D, Alduino R, et al Steatosis affects the performance of liver stiffness measurement for fibrosis assessment in patients with genotype 1 chronic hepatitis C. J Hepatol. 2014 9;61(3):523–9. 10.1016/j.jhep.2014.04.045 24815874

[26] Petta S, Maida M, Macaluso FS, Di Marco V, Cammà C, Cabibi D, et al The severity of steatosis influences liver stiffness measurement in patients with nonalcoholic fatty liver disease. Hepatology. 2015 10;62(4):1101–10. 10.1002/hep.27844 25991038

[27] Castéra L, Foucher J, Bernard PH, Carvalho F, Allaix D, Merrouche W, et al Pitfalls of liver stiffness measurement: a 5-year prospective study of 13,369 examinations. Hepatology. 2010;51(3):828–35. 10.1002/hep.23425 20063276

